# Lessons Learned From the ACURATE IDE Trial for Transcatheter Aortic Valve Replacement

**DOI:** 10.1002/ccd.70092

**Published:** 2025-08-18

**Authors:** Arturo Giordano, Giuseppe Biondi‐Zoccai, David J. Cohen, Rodrigo Bagur, Nicola Corcione

**Affiliations:** ^1^ Unità Operativa di Interventistica Cardiovascolare, Pineta Grande Hospital Castel Volturno Italy; ^2^ Department of Medical‐Surgical Sciences and Biotechnologies Sapienza University of Rome Latina Italy; ^3^ Maria Cecilia Hospital, GVM Care & Research Cotignola Italy; ^4^ Cardiovascular Research Foundation New York New York USA; ^5^ St. Francis Hospital Roslyn New York USA; ^6^ Division of Cardiology, Department of Medicine London Health Sciences Centre Western University London Ontario Canada

**Keywords:** ACURATE neo2, Boston Scientific, recall, structural heart disease, transcatheter aortic valve implantation, transcatheter aortic valve replacement

## Abstract

The recent voluntary withdrawal of the ACURATE neo2 transcatheter aortic valve replacement device by Boston Scientific offers a compelling case study in the complex interplay of device design, clinical evidence, regulatory requirements, and market dynamics in modern structural heart interventions. Despite promising performance in European and Canadian registries, the ACURATE neo2 valve failed to demonstrate non‐inferiority compared with commercially available balloon‐expandable and self‐expanding platforms in the pivotal ACURATE IDE randomized controlled trial. These results, coupled with introduction of a new regulatory requirements by the European notified body ultimately led to the global discontinuation of the platform. This review critically examines the technological characteristics of ACURATE neo2, compares it with other leading TAVR devices, and explores the potential reasons—ranging from clinical to strategic—that may have led to its market exit. Emphasis is placed on the role of randomized trials in assessing new structural therapies, including a discussion of methodological challenges and opportunities for adaptive trial designs. A structured comparison of device features and withdrawal rationales is also provided, highlighting lessons relevant to clinicians, regulators, and industry stakeholders. Ultimately, the ACURATE neo2 experience underscores the need for robust validation strategies, procedural standardization, and adaptive development pathways in a saturated and high‐stakes market. Lessons learned from this platform should inform future innovation in TAVR and broader cardiovascular device development.

## 1 Introduction

Transcatheter aortic valve replacement (TAVR) has transformed the management of severe aortic stenosis, offering a minimally invasive alternative to surgical valve replacement [1]. Over the past two decades, multiple valve platforms have emerged, each aiming to optimize procedural safety, valve durability, and hemodynamic performance. Within this dynamic landscape, the ACURATE neo valve was originally developed by Symetis SA, a Swiss start‐up company later acquired by Boston Scientific, culminating in commercial launch of the ACURATE neo2—a CE‐marked, self‐expanding valve positioned as a competitor to well‐established devices such as the SAPIEN 3 (Edwards Lifesciences) and Evolut FX/FX+ (Medtronic) [2, 3, 4, 5]. The promise of intuitive deployment, supra‐annular design, reliable coronary access and favorable hemodynamics made it appealing to TAVR operators, leading to a substantial uptake in Europe, while less so in Canada and other healthcare systems [5, 6]. However, the journey of the ACURATE neo2 ultimately illustrates the complexities of global device development, especially when transitioning from Conformité Européene (CE) mark to US Food and Drug Administration (FDA) approval [7]. This review synthesizes clinical, regulatory, and strategic lessons from the ACURATE neo2 experience, offering insights into what defines success and failure in structural heart innovation.

## 2 Device Overview: ACURATE neo2 Technology

The ACURATE neo2 valve is a second‐generation self‐expanding transcatheter aortic valve system engineered for supra‐annular function porcine pericardial leaflets. The device is designed to provide tactile feedback during top‐down positioning and deployment, anatomically aligning implantation to allow for precise deployment [4, 8].

Compared with its earlier generation device, the ACURATE neo2 incorporates an active sealing skirt (PVseal), which is designed to reduce paravalvular leak by enhancing annular conformity and sealing [9]. While the ACURATE neo2 does not allow full resheathing or retrieval, it does permit partial repositioning before final deployment. This design aims for simplicity but may offer less procedural flexibility compared to systems that allow for full resheathing and recapture during deployment. It is available in three sizes (Small: 23 mm, Medium: 25 mm, Large: 27 mm) and is compatible with the 14 French iSleeve sheath (Boston Scientific). The newest generation ACURATE Prime platform increased the size range to include the XL (29 mm) device.

The first study of the ACURATE platform was completed in 2009 leading to CE mark in 2011, and the pivotal trial of the ACURATE neo was reported in 2012, followed by CE mark in 2014. After redesign, the ACURATE neo2 was CE‐marked in 2022 and adopted in Europe, Canada, and Latin America, although it never received US FDA approval [4, 10].

## 3 Comparative Device Landscape: How Does ACURATE neo2 Measure Up?

Among contemporary TAVR devices, the ACURATE neo2 has a unique design characterized by a top‐down, self‐expanding deployment mechanism and supra‐annular leaflet function, and comprises three stabilization arches for axial alignment, an upper‐crown for capping the aortic leaflets, and a lower‐crown that is deployed within the native aortic valve (Table 1). These design features allow the device to achieve excellent hemodynamic performance while mitigating the risk of coronary obstruction and maintaining future coronary access [11]. When deployed in this fashion, ACURATE neo2 protrudes only minimally into the left ventricular outflow‐tract, thereby minimizing the risk of conduction abnormalities. Compared with balloon‐expandable platforms such as SAPIEN 3 from Edwards Lifesciences, ACURATE neo2 is associated with lower transvalvular gradients but may be more prone to paravalvular leak, especially in borderline annuli or elliptical anatomies [1, 12]. While Evolut FX+ also features a supra‐annular design, it benefits from full recapturability and repositioning, the ACURATE neo2 allows for limited repositioning only during the early phase of deployment (knob 1) and lacks full recapturability [13]. The sealing mechanism of ACURATE neo2, though improved with the PVseal skirt, can vary in relation to annular geometry as well as location and extent of leaflet and annular calcification. In contrast to current practice with both the SAPIEN and Evolut platforms, ACURATE neo2 generally requires predilation with a nominal sized balloon to optimize valve expansion and sealing and making procedural training and device‐specific experience particularly relevant. Devices like Navitor and Myval offer competitive profiles with flexible delivery systems and more extensive sizing matrices, further challenging the market positioning of ACURATE neo2 in certain patient subsets [14, 15].

**TABLE 1:** Comparative features of selected transcatheter aortic valve replacement devices.

Feature	ACURATE neo2 (Boston Scientific)	Evolut FX + (Medtronic)	Myval Octapro (Meril)	Navitor (Abbott)	SAPIEN 3 Resilia (Edwards)	VitaFlow Liberty (MicroPort)
Valve Type	Self‐expanding	Self‐expanding	Balloon‐expandable	Self‐expanding	Balloon‐expandable	Self‐expanding
Frame Material	Nitinol	Nitinol	MP35N Cobalt alloy	Nitinol	Cobalt‐chromium	Nitinol
Leaflet Position	Supra‐annular	Supra‐annular	Intra‐annular	Intra‐annular	Intra‐annular	Supra‐annular
Leaflet Material	Bovine pericardium	Porcine pericardium	Bovine pericardium	Bovine pericardium	Bovine pericardium	Bovine pericardium
Deployment Mechanism	Top‐down (self‐expanding)	Bottom‐up (self‐expanding)	Balloon expansion	Controlled expansion	Balloon expansion	Motorized, retrievable deployment
Repositionability	Partial (Step 1 only)	Yes	No	Yes	No	Partial
Resheathability	No	Yes	No	Yes	No	No
Retrievability	No	Yes	No	Yes	No	No
Valve Sizes (mm)	23, 25, 27 (29 only for ACURATE Prime)	23, 26, 29, 34	20–32 (in 1.5 mm increments)	23, 25.5, 27, 29, 31	20, 23, 26, 29	20, 23, 26, 29
Annulus Range (mm)	21–27	18–30	18–32	19–30	18–29	20–29
Sealing Technology	Active PVseal skirt	Pericardial wrap	Dual PET skirts	NaviSeal active adaptive seal	PET outer skirt	Dual‐layer adaptive skirt
Coronary Access	Excellent (open‐cell design)	Challenging (dense frame)	Favorable	Favorable	Moderate (frame height)	Favorable
Delivery Sheath (French)	14 iSleeve	14 and 18 equivalent InLine Sheath	14–16	14	14 and 16 eSheath	14–16 equivalent
Approved Markets	CE (EU, LatAm), Health Canada; not FDA‐approved	FDA, Health Canada, CE, global	CE (select markets); not FDA and Health Canada	FDA, Health Canada, CE	FDA, CE, Health Canada, global	China, CE (limited EU)
Strengths	Low pacemaker rate, negligible risk of coronary obstruction, good hemodynamics, flexible delivery system, especially for horizontal aortas	Repositionable, favorable longest term clinical and echocardiographic data, strong radial force (i.e. for heavily calcified or bicuspid valves)	Fine sizing options, ease of delivery, excellent performance in small and large annuli, excellent control with steerable delivery system	Repositionable, low gradients	Excellent control with steerable delivery system, favorable long‐term clinical and echocardiographic data	Retrievable, repositionable, strong radial force (eg heavily calcified or bicuspid valve), promising PVL control
Limitations	Requires precise technique; not resheathable or retrievable	higher PPI	Limited durability data, restricted availability in some market	Long‐term data still emerging, higher rates of PPI	Higher gradients, valve thrombosis	Limited durability and Western data, early clinical experience

Abbreviations: AR, aortic regurgitation; CE, Conformité Européenne; EU, European Union; FDA, Food and Drug Administration; PET, polyethylene terephthalate; PPI, permanent pacemaker implantation; PVL, para‐valvular leak; VIV, valve‐in‐valve.

## 4 Clinical Evidence: From Registries to the ACURATE IDE Trial

Clinical data on the ACURATE neo2 valve initially appeared promising, with European studies such as NEOPRO and Early neo2 suggesting favorable hemodynamic profiles and acceptable safety outcomes [7, 16]. However, optimism waned following the ACURATE Investigational Device Exemption (IDE) trial—a randomized controlled trial (RCT) comparing ACURATE neo2 to other FDA‐approved TAVR platforms [17]. The trial failed to meet its primary non‐inferiority endpoint as treatment with ACURATE neo2 led to numerically higher rates of all‐cause mortality, stroke, and rehospitalization at 1 year compared with the control valves (14.8% vs 9.1%, hazard ratio of 1.71 [95% confidence interval 1.26–2.33]; *p* = 0.0005). Subsequent post‐hoc analyses pointed to procedural issues such as underexpansion and inconsistent use of pre/post‐dilatation as potential contributing factors [18]. The divergence between registry and RCT findings may reflect differences in patient selection, operator training, procedural technique, and overall learning curve.

## 5 Root Causes of Trial Underperformance

One of the pivotal factors contributing to the underperformance of the ACURATE IDE trial appears to have been the high rate of valve underexpansion, observed in nearly 20% of implanted cases [17, 18]. This mechanical shortcoming likely compromised leaflet coaptation and contributed to higher than expected rates of thromboembolic events (i.e., myocardial infarction and stroke) as well as paravalvular leak and early valve dysfunction. Operator‐related variability, especially in centers with limited prior experience with the device, may have further exacerbated procedural inconsistency and suboptimal deployment. Although the ACURATE neo2 lacks full repositionability, this was likely not the primary driver of adverse outcomes—particularly since most patients in the control group received SAPIEN balloon‐expandable valves that also lack this feature. Also important, SAPIEN is implanted without predilation in most cases as shown in the ACURATE IDE trial, and operators might have relied, probably too much, on their experience with SAPIEN and this has ultimately impacted on the procedural aspects exposed with the use of ACURATE neo2. Finally, trial execution may have been impacted the outcomes of the ACURATE neo2, mainly because of limited operator experience with the device and lack of procedural standardization, particularly regarding systematic predilation and proper balloon sizing. Collectively, these factors are likely to have undermined the trial's ability to demonstrate non‐inferiority compared with established platforms like SAPIEN 3 and Evolut. These insights highlight the critical importance of integrating robust pre‐trial training, device‐specific implantation protocols, and anatomical selection algorithms in pivotal device studies—even when the procedure, itself, is well‐established.

## 6 Regulatory and Strategic Fallout

The regulatory and strategic fallout following the ACURATE IDE trial marks a pivotal inflection point for the Boston Scientific TAVR program. The failure to achieve non‐inferiority in the trial prompted the company to halt its pursuit of FDA approval, effectively ending its US foray into this space. As a result, Boston Scientific announced the global discontinuation of the whole ACURATE neo2 program. According to Reuters, Boston Scientific said that “recent discussions with U.S. and other regulators resulted in increased requirements for approvals, and the resources and investments necessary to meet them were prohibitive. The requirements included the need for a new randomized clinical trial, establishment of registries and other extensive post‐market surveillance activities.” [16] An additional factor in this decision was the imposition of additional regulatory hurdles by the European notified body to maintain CE mark status—a demand that Boston Scientific considered commercially unsustainable. This decision led to internal restructuring, including layoffs in the structural heart division and reallocation of research and development budgets [19]. Strategically, it reflects a broader reorganization, favoring higher‐certainty pipelines and divesting from high‐risk, late‐stage devices lacking a clear competitive advantage or regulatory path forward.

## 7 Understanding the Decision to Withdraw ACURATE Neo2 From the Market

Since no manufacturing defects have been reported with the ACURATE neo2 device, it seems likely that prevailing market dynamics—dominated by well‐established TAVR competitors—were a major factor in the decision to withdraw the device from the global market. Table 2 summarizes the hierarchy of clinical, regulatory, and commercial factors that likely contributed to Boston Scientific's strategic decision. The ACURATE experience thus stands as a cautionary tale in the high‐stakes landscape of structural heart innovation, reinforcing the importance of multiple factors including robust trial design, device‐specific procedural standardization, and continuous performance evaluation throughout the premarket pathway.

**TABLE 2:** Potential factors influencing the decision to withdraw the ACURATE neo2 transcatheter aortic valve replacement device (TAVR).

Likelihood	Reason	Evidence/Reviewer‐Aligned justification
Most Likely	Disproportionate regulatory demands from European notified body	Requirement for 100% follow‐up of all commercial ACURATE Prime cases in Europe to maintain CE mark status rendered continued commercial development unsustainable.
	Failure to demonstrate non‐inferiority in pivotal trial (IDE)	ACURATE neo2 did not meet primary endpoint of non‐inferiority versus control; higher stroke and mortality rates documented despite good hemodynamics.
Likely	Valve underexpansion and procedural sensitivity	Post‐hoc analysis of the IDE trial revealed ~20% rate of underexpansion, often associated with suboptimal sizing and deployment technique.
	Operator learning curve and procedural variability	IDE trial outcomes varied widely across centers, suggesting influence of familiarity, case selection, and early‐phase experience.
Possible	Intense market competition	Edwards (SAPIEN) and Medtronic (Evolut) dominate TAVR market; newer entrants (e.g., Myval) offer simplified platforms at lower cost.
Unlikely	Device design or manufacturing deficiencies	No widespread quality control alerts issued.

Abbreviation: IDE, investigational device exemption.

## 8 Broader Lessons for Device Innovation

The ACURATE neo2 experience underscores the critical importance of aligning technical innovation with rigorous procedural validation. While some design elements—such as the PVseal skirt and supra‐annular function—offered clinically meaningful advantages over competing platforms (e.g., low gradients, low risk of coronary obstruction, and pacemaker rates in the low single digits, their real‐world effectiveness may have been confounded by operator learning curves, and procedural planning variability). Failure to demonstrate non‐inferiority in the pivotal RCT also highlights the risks of advancing prematurely without broad procedural familiarity or strict adherence to technical recommendations. This case thus illustrates how even CE‐marked technologies with broad commercial acceptance may falter under the scrutiny of global regulatory standards and randomized trials. Future device development must integrate robust training and multicenter pilot studies to ensure procedural proficiency and consistency. Moreover, iterative feedback from early adopters and registries should inform design refinements before large‐scale rollout [20]. Ultimately, innovation in structural heart therapies must balance novelty with reproducibility, safety, and real‐world adaptability.

## 9 The Indispensable Role of RCTs in Structural Heart Therapies

High‐quality RCTs remain the foundation of evidence in structural heart interventions, offering rigorous assessments that real‐world registries alone cannot provide [19, 21]. The ACURATE IDE trial illustrated this, uncovering safety and efficacy limitations not readily apparent in earlier observational data. Despite favorable hemodynamics in small studies, in the pivotal trial, the device failed to meet non‐inferiority for the composite endpoint of death, stroke, or rehospitalization—highlighting how operator learning curve, procedural variability, and trial implementation can influence outcomes. Future clinical trials could incorporate a prolonged single‐arm “roll in” phase during which inexperienced centers would accumulate critical experience with the device, with randomization allowed only after a defined number of successful, uncomplicated cases has been accrued and using similar trial endpoints. As next‐generation TAVR platforms target increasingly complex anatomies, integrating simulation tools, AI‐based modeling, and real‐world feedback into trial design will be essential. The ACURATE neo2 experience reaffirms that high‐quality comparative data and innovative trial methodologies are critical for both regulatory success and safe clinical adoption.

## 10 Implications for TAVR Industry Evolution

**FIGURE 1:**
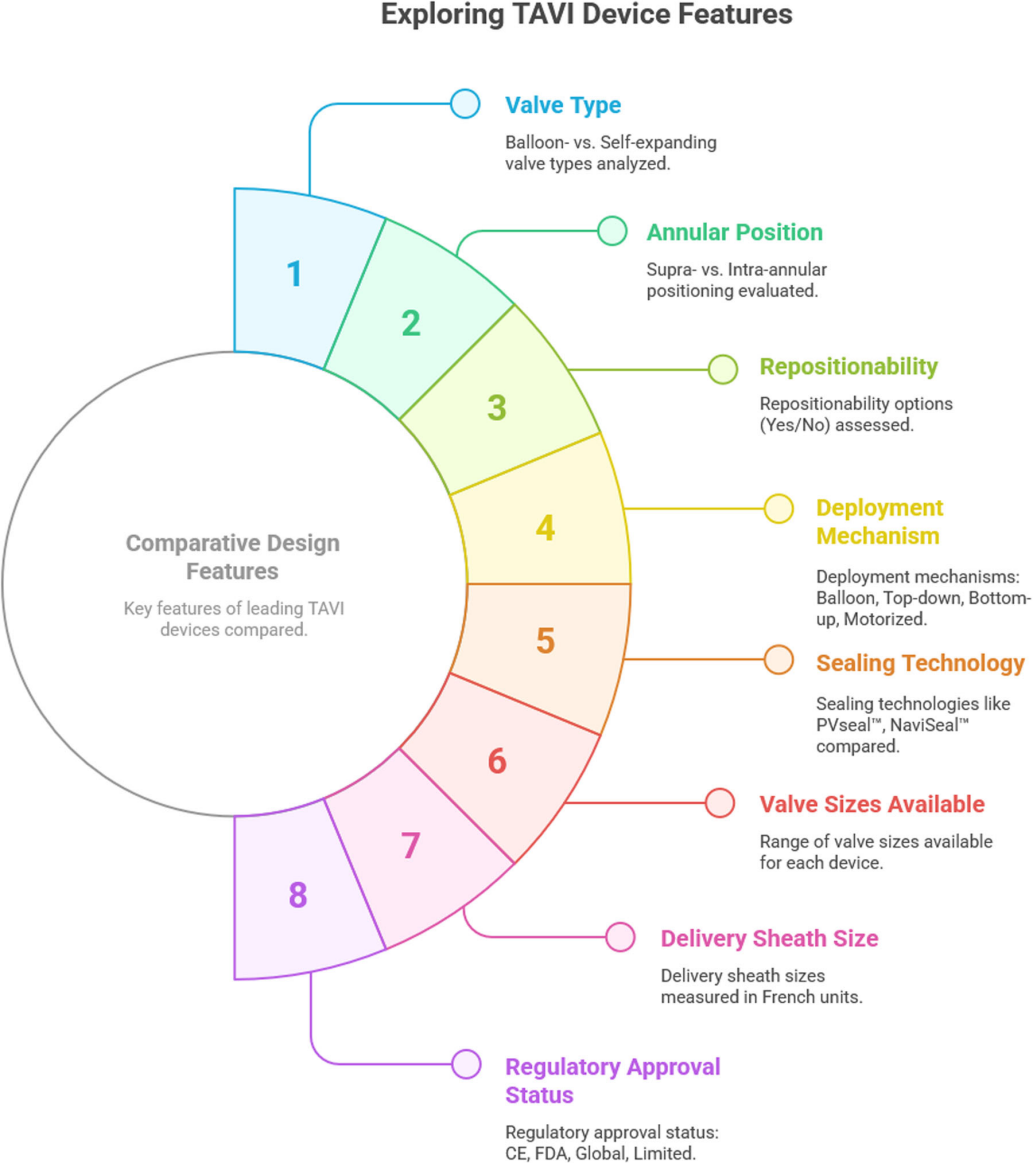
Multidimensional appraisal of transcatheter aortic valve replacement devices. [Color figure can be viewed at wileyonlinelibrary.com]

The withdrawal of ACURATE platform underscores the intensifying pressures in the TAVR market, where only platforms that demonstrate clear value through durable outcomes, procedural consistency, and strong regulatory alignment can thrive (Figure 1). As the field matures, regulatory scrutiny and clinical expectations are rising, leaving little room for platforms that fail to meet evolving performance and surveillance standards. Consolidation among dominant players—Abbott, Medtronic and Edwards—suggests a trend toward fewer but more refined solutions. Future TAVR devices will likely emphasize ease of use, coronary access preservation, and adaptive design for reintervention [22]. Moreover, enhanced delivery systems and anti‐thrombogenic surfaces may become standard. The ACURATE saga reinforces that innovation alone is insufficient without consistent reproducibility, procedural forgiveness, and sustainable post‐market evidence generation [23]. For industry stakeholders, the message is clear: survival demands excellence on all fronts, and adaptability to both clinical complexity and regulatory stringency.

## 11 Conclusions

The history of ACURATE platform stands as a vivid case study in the complex interplay between device design, clinical performance, regulatory demands, and market forces. Despite promising engineering and early registry data, the inability of the ACURATE neo2 valve to meet pivotal trial endpoints proved decisive in halting its global trajectory. Boston Scientific's subsequent withdrawal from the TAVR space highlights how even well‐established companies can falter in a highly competitive and scrutinized field. The episode also underscores the fundamental role of rigorous randomized controlled trials in validating new technologies and demonstrates the consequences of underestimating procedural complexity, anatomical variability, and the demands of operator training and device‐specific experience. Ultimately, the ACURATE neo2 story offers important lessons for innovators, emphasizing the need for robust pre‐market evidence, adaptable development strategies, balanced assessment of device‐specific trade‐offs, and transparent post‐market reassessment. The case also underscores how strict regulatory expectations can critically influence commercial viability.

## Conflicts of Interest

Giuseppe Biondi‐Zoccai has consulted, lectured and/or served as advisory board member for Abiomed, Advanced Nanotherapies, Aleph, Amarin, AstraZeneca, Balmed, Cardionovum, Cepton, Crannmedical, Endocore Lab, Eukon, Guidotti, Innovheart, Meditrial, Menarini, Microport, Opsens Medical, Synthesa, Terumo, and Translumina, outside the present work. David Cohen reports consulting income from Abbott, Boston Scientific, Edwards Lifesciences, Elixir Medical Medtronic, and ZOLL Medical and also institutional research grant supports from Abbott, Boston Scientific, Corcinch, Corvia, Edwards Lifesciences, Philips, and ZOLL Medical. Rodrigo Bagur is consultant and proctor for Medtronic and works in an institution that was training centre for Acurate neo2 in North America, also enrolling patients in the ACURATE IDE Pivotal trial. All other authors report no conflict of interest.
